# Supplementary material for: Face mask use in the city of Chennai, India: Results from three serial cross-sectional surveys, 2021
Source: PLoS One. 2024 Apr 4;19(4):e0297909. doi: 10.1371/journal.pone.0297909 (PMC10994359; doi:10.1371/journal.pone.0297909)
Supplement: S1 File — (PDF) [file pone.0297909.s001.pdf]

Supplementary File 1 for the manuscript

Face mask use in the city of Chennai, India: Results from three serial cross-sectional surveys, 2021

S1 Table: Compliance with appropriate face mask usage by slum and non-slum in the exposure categories (age group, gender, and region) for the three rounds in the outdoor study locations, Chennai, India

S2 Table: Compliance with appropriate face mask usage by slum and non-slum in the exposure categories (age group, gender, and region) for the three rounds in the indoor study locations, Chennai, India

S3 Table: Compliance with appropriate face mask usage in the shopping malls by the exposure categories (age group and gender) in the fifth round of mask study, Chennai, India

S1 Table: Compliance with appropriate face mask usage by slum and non-slum in the exposure categories (age group, gender, and region) for the three rounds in the outdoor study locations, Chennai, India

Characteristic		20 - 26 March 2021 (Round 3)					8 -10 July 2021 (Round 4)					30 Oct - 1 Nov 2021 (Round 5)				
		Slum (N=1600)					Slum (N=1600)					Slum (N=1600)				
		N	n	(%)	95% CI	p-value	N	n	(%)	95% CI	p-value	N	n	(%)	95% CI	p-value
**Age (Years)**	Children/ adolescent	138	13	(9)	5 - 17		139	39	(28)	14 - 42		95	21	(22)	9 - 45	
	Young adult	482	124	(26)	20 - 33	**<0.001**	577	270	(47)	39 - 55	**<0.001**	445	180	(40)	35 - 46	**<0.001**
	Middle-aged	747	164	(22)	17 - 28		677	282	(43)	35 - 51		829	271	(33)	28 - 38	
	Older	233	43	(19)	13 - 26		207	49	(24)	15 - 32		231	46	(20)	13 - 30	
**Sex**	Male	1147	267	(23)	18 - 30	**0.006**	1178	515	(44)	37 - 51	**<0.001**	1230	410	(33)	29 - 38	**0.001**
	Female	453	77	(17)	11 - 25		422	131	(32)	23 - 39		370	108	(29)	23 - 37	
**Region**	North	800	113	(14)	9 - 22		800	328	(41)	32 - 51		800	262	(33)	28 - 38	
	Central	500	162	(32)	25 - 41	**<0.001**	500	177	(36)	23 - 48	**0.018**	500	152	(30)	20 - 43	0.231
	South	300	69	(23)	15 - 33		300	141	(47)	34 - 60		300	104	(35)	22 - 50	
**Overall**		**1600**	**344**	**(22)**	**17 - 27**		**1600**	**646**	**(41)**	**34 - 47**		**1600**	**518**	**(32)**	**28 - 37**	

Characteristic		Non-Slum (N=1600)					Non-Slum (N=1600)					Non-Slum (N=1600)				
		N	n	(%)	95% CI	p-value	N	n	(%)	95% CI	p-value	N	n	(%)	95% CI	p-value
**Age (Years)**	Children/ adolescent	159	26	(16)	11 - 24		118	40	(34)	24 - 44		81	24	(30)	19 - 43	
	Young adult	455	140	(31)	24 - 38	**<0.001**	578	306	(53)	46 - 59	**<0.001**	444	183	(41)	34 - 49	**<0.001**
	Middle-aged	733	215	(29)	24 - 35		687	341	(50)	43 - 56		825	279	(34)	28 - 40	
	Older	253	54	(21)	14 - 31		217	80	(37)	29 - 44		250	77	(31)	23 - 41	
**Sex**	Male	1263	355	(28)	24 - 33	0.164	1183	570	(48)	43 - 53	0.778	1270	445	(35)	30 - 41	0.859
	Female	337	80	(24)	17 - 32		417	197	(47)	40 - 56		330	118	(36)	27 - 46	
**Region**	North	350	61	(17)	7 - 39		350	184	(53)	39 - 66		350	112	(32)	22 - 45	
	Central	550	162	(29)	23 - 37	**<0.001**	550	261	(48)	39 - 56	0.359	550	257	(47)	37 - 57	**<0.001**
	South	700	212	(30)	24 - 38		700	322	(46)	40 - 52		700	194	(28)	21 - 35	
**Overall**		**1600**	**435**	**(27)**	**23 - 33**		**1600**	**767**	**(47)**	**43 - 53**		**1600**	**563**	**(35)**	**29 - 41**	

S2 Table: Compliance with appropriate face mask usage by slum and non-slum in the exposure categories (age group, gender, and region) for the three rounds in the indoor study locations, Chennai, India

Characteristic		20 - 26 March 2021 (Round 3)					8 -10 July 2021 (Round 4)					30 Oct - 1 Nov 2021 (Round 5)				
		Slum (N=640)					Slum (N=640)					Slum (N=640)				
		N	n	(%)	95% CI	p-value	N	n	(%)	95% CI	p-value	N	n	(%)	95% CI	p-value
Age (Years)	Children/ adolescent	38	5	(13)	5-31		31	10	(32)	15-56		36	11	(31)	12-58	
	Young adult	178	25	(14)	8-22	0.032	176	46	(26)	18-36	0.251	128	23	(18)	10-29	<0.001
	Middle-aged	312	31	(10)	6-15		332	71	(21)	15-30		364	42	(12)	8-17	
	Older	112	11	(10)	3-26		101	26	(26)	17-37		112	16	(14)	7-26	
Sex	Male	475	54	(11)	8-17	0.950	461	116	(25)	19-33	0.212	432	54	(13)	8-18	<0.001
	Female	165	18	(11)	6-19		179	37	(21)	14-30		208	38	(18)	9-33	
Region	North	320	33	(10)	6-18		320	58	(18)	12-27		320	30	(9)	5-17	
	Central	200	30	(15)	7-29	0.062	200	50	(25)	14-40	0.001	200	34	(17)	7-34	0.001
	South	120	9	(8)	4-13		120	45	(38)	20-58		120	28	(23)	5-62	
Overall		640	72	(11)	8-16		640	153	(24)	18-31		640	92	(14)	9-22	

Characteristic		Non-Slum (N=640)					Non-Slum (N=640)					Non-Slum (N=640)				
		N	n	(%)	95% CI	p-value	N	n	(%)	95% CI	p-value	N	n	(%)	95% CI	p-value
Age (Years)	Children/ adolescent	47	4	(9)	3-22		21	6	(29)	11-56		28	10	(36)	18-58	
	Young adult	171	35	(21)	15-28	<0.001	240	91	(38)	28-49	0.047	153	28	(18)	11-29	0.002
	Middle-aged	304	48	(16)	12-21		295	87	(29)	23-37		310	60	(19)	14-26	
	Older	118	17	(14)	9-23		84	28	(33)	25-41		149	37	(25)	17-36	
Sex	Male	475	74	(16)	12-20	0.669	477	164	(34)	28-41	0.145	473	82	(17)	13-23	<0.001
	Female	165	30	(18)	11-29		163	48	(29)	19-43		167	53	(32)	23-42	
Region	North	140	17	(12)	6-23		140	40	(29)	15-48		140	19	(14)	7-26	
	Central	220	43	(20)	12-30	0.068	220	87	(40)	26-54	0.025	220	56	(25)	16-38	0.017
	South	280	44	(16)	11-23		280	85	(30)	22-40		280	60	(21)	14-32	
Overall		640	104	(16)	13-21		640	212	(33)	27-40		640	135	(21)	16-27	

S3 Table: Compliance with appropriate face mask usage in the shopping malls
by the exposure categories (age group and gender) in the fifth round of mask study, Chennai, India

Characteristic		30 Oct - 1 Nov 2021 (Round 5)				
		Malls (N=1650)				
		N	n	(%)	95% CI	p-value
**Age (Years)**	Children/ adolescent	109	66	(60)	43 - 76	**0.039**
	Young adult	787	454	(58)	50 - 65	
	Middle-aged	635	340	(54)	42 - 64	
	Older	119	75	(63)	47 - 77	
**Sex**	Male	1034	563	(54)	44 - 64	0.061
	Female	616	372	(60)	53 - 67	
**Overall**		1650	935	(57)	48 - 65	
