# Supplementary material for: Face mask use in the city of Chennai, India: Results from three serial cross-sectional surveys, 2021
Source: PLoS One. 2024 Apr 4;19(4):e0297909. doi: 10.1371/journal.pone.0297909 (PMC10994359; doi:10.1371/journal.pone.0297909)
Supplement: Attachment — Submitted filename: Response to Revieweres 2.pdf [file pone.0297909.s002.pdf]

No.	Comment from	Comment	Response
1	Academic Editor	(a) Please clarify the sources of funding (financial or material support) for your study. List the grants or organizations that supported your study, including funding received from your institution.<br><br>(b) State what role the funders took in the study. If the funders had no role in your study, please state: "The funders had no role in study design, data collection and analysis, decision to publish, or preparation of the manuscript."<br><br>(c) If any authors received a salary from any of your funders, please state which authors and which funders.<br><br>(d) If you did not receive any funding for this study, please state: "The authors received no specific funding for this work"	The Authors have received no specific funding for this study. The authors have received only material support from their organisation Indian Council of Medical Research - National Institute of Epidemiology (ICMR NIE), Chennai, India. The organization had no conflict of interest with the study. ICMR NIE had no role in study design, data collection and analysis, decision to publish, or preparation of the manuscript.
2	Academic Editor	In your Data Availability statement, you have not specified where the minimal data set underlying the results described in your manuscript can be found. PLOS defines a study's minimal data set as the underlying data used to reach the conclusions drawn in the manuscript and any additional data required to replicate the reported study findings in their entirety. All PLOS journals require that the minimal data set be made fully available. For more information about our data policy, please see http://journals.plos.org/plosone/s/data-availability.	The minimal dataset required for reproducing the findings from the paper is fully available in the following citation:<br><br>Murali, Sharan; Sakthivel, Manikanda Nesan; Rubeshkumar, Polani Chandrasekar; Nagarajan, Ramya; Venkatasamy, Vettrichelvan; Muthappan, Sendhilkumar; et al.(2023), "Face mask use in the city of Chennai, India: Results from three serial cross-sectional surveys, 2021", Mendeley Data, V1, doi: 10.17632/vkv4cwhh7n.1
3	Academic Editor	We note that Figure 1 in your submission contain map images which may be copyrighted. All PLOS content is published under the Creative Commons Attribution License (CC BY 4.0), which means that the manuscript, images, and Supporting Information files will be freely available online, and any third party is permitted to access, download, copy, distribute, and use these materials in any way, even commercially, with proper attribution. For these reasons, we cannot publish previously copyrighted maps or satellite images created using proprietary data, such as Google software (Google Maps, Street View, and Earth).	We have used the map in Figure 1 to demonstrate the spread of the locations of our study. We have used the shape file available in the open domain to create this map. The map was downloaded from the website: https://data.opencity.in/dataset/gcc-ward-information.<br><br>We have updated the caption for Figure 1 in the manuscript file. We have also added the reference.<br><br>Since this is open-source and publicly available, we believe copyright issues won't exist.
4	Reviewer 1	Kindly rewrite the abstract, as the methods mentioned in the abstract is confusing, and please stick to the abstract guidelines specific for Plos One	Thanks for the suggestion. We have rewritten the abstract now to bring more clarity to the methods section. It also aligns with the guidelines for abstracts in Plos One and has 293 words in a structured format. (Lines 26 to 30)
5	Reviewer 1	Line 73, which reads "We also documented mask compliance in the city's shopping malls for the third time point (round 5) alone" is not appropriately placed and is confusing. How many rounds were conducted? 3 or 5? Please be specific and modify the statement.	Thank you for bringing this issue up. We have now corrected this line to bring out the idea more correctly. We have completed 5 surveys in Chennai during the COVID-19 period. Two initial surveys conducted in 2020 were published in Plos One - Reference no. 12. This manuscript talks about the findings in the last three rounds - Rounds 3,4, and 5. We have also added an objective where compliance is compared across the five-time points. (line 73-75)
6	Reviewer 1	Why mention about rounds 1 and 2 from 2020 in this study? when the objective is to estimate compliance with appropriate mask usage at three different time points in 2021?	This manuscript is in continuation to the rounds 1 and 2 that we conducted in 2020. This paper exclusively brings out the methods and results which we have conducted in 2021. We use the data for 2020 to compare and quantify the trend in compliance with time. We have now added this as objective 3. (Line 73-75)
7	Reviewer 1	Why were these specific time periods chosen for sampling? the justification for the final round is provided, please provide the rationale and justification for conducting the survey at specific time periods of 2021	We have added justification to choosing each of the periods for each of the rounds. The surveys were conducted in line with the second COVID-19 wave and as per the instructions received from the Greater Chennai Corporation. (Line 80 -86).
8	Reviewer 1	Why were 32 streets chosen? and how were the 32 chosen from 45,000? what sampling strategy was followed?	Thank you for the suggestion. The sampling strategy was missing in the text. We have now explained the same in lines 97-100.<br><br>We have added the sampling strategy for the indoor settings also. (Line 104-106)
9	Reviewer 1	Pharmacies and religious places had "no entry restrictions?" and thus considered indoor settings.? not even in pharmacies? a personal would insist on mask usage?	We understand that this needs a little more clarity. Pharmacies, shops, and religious places in Chennai had no personnel to insist on mask usage. And the responsibility of wearing the mask is entirely on the individual entering the place. Thus, we included these locations as indoor spaces. We have rewritten this context in the manuscript now. Line 120 - 127
10	Reviewer 1	"We excluded areas with security personnel who insisted on wearing a mask before entry.. Then why include Malls? which mandates mask usage before entry?	In continuation to the previous point raised, Malls had personnel to insist on mask usage. But this checking was only at the entrance. So once the checking is done, again the mask-wearing responsibility is on the individual. Therefore we considered malls as a separate entity from Indoor locations. Line 126-127

No.	Comment from	Comment	Response
11	Reviewer 1	How was it possible to record the mask usage and compliance among motorcycle riders, who wear helmets?	Thank you for the suggestion. We had excluded the riders who were wearing a helmet. This detail was missing in the version submitted. We have corrected this error as below:<br><br>We included pedestrians, bicycle riders, motorcycle riders, autorickshaws, and bus passengers, excluding individuals traveling in a car, and riders wearing a helmet. (line 138-139)
12	Reviewer 1	How did the estimates calculate for the population inside the moving bus? how did the data collector collect this information? Were they stationed in the street or inside the bus?	Our study did not aim to estimate the compliance for mask usage at the population level inside a bus. We aimed to quantify the proportion of individuals who have worn the mask appropriately in the outdoor setting - which includes the vehicles that passed by. So the Stationary data collector will watch for one individual at a time (walking, stationary or inside the bus) and note the findings. The data collector would then look-up and choose another individual within their vicinity. This process was repeated till the required sample size in the setting was achieved. The data collector didnot get into any moving vehicles.
13	Reviewer 1	Table 1: Round 3 is March 2020? or 2021?	Thanks for bring this typographical error to our notice. Its March 2021, We have now corrected the same
14	Reviewer 1	When the objective is not to compare with the 2020 estimates, I feel there is no necessity to compare with round 1 and 2 estimates.	Thank you for bringing this comment. Considering that the study is to document the mask compliance and to see if its changing over time, we thought we will compare with the 2020 surveys as well. This comparison will allow us to see if with time (as pandemic goes on) were individuals adopting mask more or vice versa. We understand that this was not very clearly written in the introduction part. We have now added it to bring more clarity (Line 73-75)
15	Reviewer 1	Please check the referencing style.. eg 2,10,13 are not in Vancouver	The references 2,10,13 have been rewritten as per the journal guidelines.
16	Reviewer 1	Please provide clearer images, preferably in TIFF format	All images are submitted in TIFF Format for the production.
17	Reviewer 1	Why did the study observe higher compliance during July 2021, while the peak was in March - April 2021?	Thank you sir for mentioning this. Yes, this is a finding from our study and the same is discussed in line 251-253.
18	Reviewer 2	The line number 71, which reads “We estimated the compliance with appropriate mask usage in the outdoor and indoor locations of slums and non-slums of Chennai, Tamil Nadu, India, at three different time points in 2021.We also documented mask compliance in the city's shopping malls for the third time point (round 5) alone.” Please modify the statements for clarification and justify the inclusion of shopping malls in the survey.	Thank you for the suggestion. We have rewritten the objectives of the study as below (Line 70-75)<br><br>We estimated the compliance with appropriate mask usage in the outdoor and indoor locations of slums and non-slums of Chennai, Tamil Nadu, India, at three different time points in 2021. During the last survey in 2021, we documented the mask compliance in the city's shopping malls, in addition to outdoor location and indoor spaces. We compared the trend in the mask compliance in the city during the five rounds conducted; two in 2020 and three in 2021.
19	Reviewer 2	Also, line number 118 states, “We excluded areas with security personnel who insisted on wearing a mask before entry” please justify the inclusion of shopping malls in the survey as these areas were with strict enforcement for wearing masks.	We understand that this needs a little more clarity. Pharmacies, shops, and religious places in Chennai had no personnel to insist on mask usage. And the responsibility of wearing the mask is entirely on the individual entering the place. Thus, we included these locations as indoor spaces. We have rewritten this context in the manuscript now. Line 122-127.<br><br>However, malls had the personnel to insist on mask usage. But this checking was only at the entrance. So once the checking is done, again the mask-wearing responsibility is on the individual. Therefore we considered malls as a separate entity from Indoor locations. Justification for adding malls is provided.
20	Reviewer 2	As the objective was to estimate the compliance with appropriate mask usage at three different time points in 2021, justify the comparison of findings from the round 1 and 2 surveys of 2020 in the current study.	We believe that the mask-wearing behavior would have changed from the pattern we saw in 2020 (the start of a pandemic) to 2021 (living with the pandemic), and hence have compared the findings of this paper with the previously published results of rounds 1 and 2. Thus we added it as an objective as below (lines 74-75):<br><br>We compared the trend in mask compliance in the city during the five rounds conducted; two in 2020 and three in 2021

No.	Comment from	Comment	Response
21	Reviewer 2	Line 109 mention, Mask" was defined as any cloth mask, medical mask, or N95 respirator worn over the face. We described appropriate mask use as wearing a mask covering the nose, mouth, and chin and inappropriate use as wearing the mask either below the nose or mouth. When the individual did not wear a mask, we considered it no mask. Cite reference for the same.	We have added the reference for this operational definition now. We adapted this definition from the published paper of rounds 1 and 2 (Reference No: 12).
22	Reviewer 2	Line 130, which reads, “We included pedestrians, bicycle riders, motorcycle riders, autorickshaws, and bus passengers, excluding individuals traveling in a car.” How was it feasible/possible to record the mask usage and compliance of helmet-wearing motorcycle riders? as well as the bus passengers inside the bus?	Thank you for the suggestion. We had excluded the riders who were wearing a helmet. This detail was missing in the version submitted. We have corrected this error as below:<br><br>We included pedestrians, bicycle riders, motorcycle riders, autorickshaws, and bus passengers, excluding individuals traveling in a car, and riders wearing a helmet. (line 138-139)
23	Reviewer 2	Line 145, “We classified the mask compliance in each of the four settings into three categories: appropriate, inappropriate, and no mask, and computed the proportion and 95% confidence interval (CI). But the findings on ‘inappropriate use’ and ‘no mask use’ category were not mentioned.	Thank you for bringing this to our notice. The objective of the present study is to document compliance with appropriate mask use and hence we summarised only that in our paper. We have therefore rewritten this line as:<br><br>We summarised the appropriate mask compliance in each of the four settings as proportion and 95% confidence interval (CI). (Line 153-154)<br><br>We have also made the necessary changes in the operational definition to bring consistency in terminologies.
24	Reviewer 2	Line 127 states, “Upon reaching the specific tagged location, the data collector would observe the individuals crossing from right to left direction, one individual at a time, and record the individual’s mask compliance. We maintained this directional rule to minimize possible duplication. Also mention the status of the observer as it may lead to duplication of the observation - whether stationary observer measured adherence by passersby, or a mobile observer measured adherence in stationary people.	We agree that there was a lack of clarity in this line. We have corrected this line as below:<br><br>Upon reaching the specific tagged location, the stationary data collector would observe the individuals crossing from right to left direction, one individual at a time, and record the individual's mask compliance (Line 134-136).
25	Reviewer 2	For the south region, the compliance was low in round 1 and 2 whereas it was high in round 3-5, comparing with other regions of the city. Justify the findings.	We have justified this finding in the lines 282-285:<br><br>"The southern region of Chennai, which is the hub for information technology companies, showed higher compliance when compared to that of the central and northern areas. (31). The level of education and social acceptance might play an essential role in mask-use behaviors."<br><br>Rounds 1 and 2 were conducted in the first wave when the informational technology (IT) companies were closed. So probably the native population of south Chennai would have recorded poor compliance. In 2021, the IT companies have slowly started opening up and hence would have registered higher compliance.
26	Reviewer 2	Please check for the year in Table 1 for Round 3. Was it March 2020 or 2021?	Thanks for bring this typographical error to our notice. Its March 2021, We have now corrected the same
27	Reviewer 2	Please check the reference numbers 2,10 and 13 for its styling with reference to the journal guidelines.	The references 2,10,13 have been rewritten as per the journal guidelines.
28	Reviewer 2	Mention about the generalizability of the study findings? Also mention the strengths and limitations.	The results of our study are generalizable to any metro city with varied education and occupation backgrounds. We have added a paragraph to ensure that the strengths and weakness of this study is brought out clearly. Line 286-290.<br><br>Our study was a rapid and low-cost methodology to monitor mask compliance in public places by direct in-person observation which is one of the recommended methods for measuring compliance to safe behaviors. Our study was able to give mask compliance in different environments in Chennai city – Outdoors, Indoors, and also in Shopping Malls. Our study had some limitations too. In the present study, we anticipate possible inter-observer variation in identifying the individual's age group. But through vigorous training, simulation exercises, and observation rules, we have tried to keep this bias to the minimum.
